# Alternative mRNA Splicing in the Pathogenesis of Obesity

**DOI:** 10.3390/ijms19020632

**Published:** 2018-02-23

**Authors:** Chi-Ming Wong, Lu Xu, Mabel Yin-Chun Yau

**Affiliations:** 1 Department of Health Technology and Informatics, The Hong Kong Polytechnic University, Hong Kong, China; 2 The State Key Laboratory of Pharmaceutical Biotechnology, Department of Medicine, Li Ka Shing Faculty of Medicine, The University of Hong Kong, Hong Kong, China; irisxu@hku.hk; 3 School of Medical and Health Sciences, Tung Wah College, Hong Kong, China; mabelyau@twc.edu.hk

**Keywords:** alternative mRNA splicing, obesity-related diseases, splice variants, splicing factors, insulin receptor, leptin receptor, lipin-1, nuclear receptor corepressor, LMNA, RNA binding protein, Fox-1 Homolog 2, NOVA splicing factors

## Abstract

Alternative mRNA splicing is an important mechanism in expansion of proteome diversity by production of multiple protein isoforms. However, emerging evidence indicates that only a limited number of annotated protein isoforms by alternative splicing are detected, and the coding sequence of alternative splice variants usually is only slightly different from that of the canonical sequence. Nevertheless, mis-splicing is associated with a large array of human diseases. Previous reviews mainly focused on hereditary and somatic mutations in cis-acting RNA sequence elements and trans-acting splicing factors. The importance of environmental perturbations contributed to mis-splicing is not assessed. As significant changes in exon skipping and splicing factors expression levels are observed with diet-induced obesity, this review focuses on several well-known alternatively spliced metabolic factors and discusses recent advances in the regulation of the expressions of splice variants under the pathophysiological conditions of obesity. The potential of targeting the alternative mRNA mis-splicing for obesity-associated diseases therapies will also be discussed.

## 1. Introduction

Alternative mRNA splicing plays a key role in enhancing protein diversity [1]. Based on a recent analysis of the statistics for annotated human nuclear genes in GeneBase 1.1, nearly 80% of human protein-coding genes produce more than one transcript [2]. On average, human protein-coding genes contain ~11 exons per transcript and produce 5.4 mRNAs per gene. The longest exon in human is exon 5 of the zinc finger and BTB domain containing 20 (ZBTB20) gene which has 24927 nucleotides (nt), and the shortest human exon is exon 2 of septin 7 (SEPT7) gene with only two nt [2]. Most mammalian pre-mRNAs contain short exonic sequences separated by longer intronic stretches. 

Ninety-four percent of mammalian protein-coding exons exceed 51 nucleotides (nt) in length [3]. It was a greater challenge to recognize the micro-exons (≤51 nt) than for the longer exons by the process of splicing machinery [3]. The size of human introns also varies considerably from 30 nt [intron 9 of macrophage stimulating one like (MST1L) gene] to ~1,160,000 nt [intron 2 of roundabout guidance receptor 2 (ROBO2) gene] [2]. Minimal length of intron is required for maintaining efficient splicing [4]. However, unlike the exons, short introns (>100 nt) are common in human transcriptome [5,6]. It is noted that short introns promote the generation of protein isoforms and they are often alternatively spliced [6].

Existence of introns is still one of the biggest mysteries in biology. Huge energy is spent on the production of introns and maintenance of the highly accurate cleavage/ligation mechanism. It is a heavy burden on cells. It is generally accepted that alternative mRNA splicing of exons is mainly for the production of multiple protein isoforms from the same gene [1]. The development of high-throughput array and sequencing technologies entirely revolutionized the analysis of alternative splicing events by allowing an unbiased assessment [7]. Remarkably, only a limited number of annotated alternative isoforms are detected in proteomics studies [8]. In other words, despite multiple alternative mRNA transcripts per gene are detected, most genes encode only one protein isoform under most circumstances [9]. The phenomenon may be attributed to the rare expression of alternative mRNA transcripts that express only in particular tissues and developmental stages [10,11]. 

It is also hypothesized that alternative mRNA splicing increases the chance of removal of the important domains for modulating the protein-protein interactions. About 70% of the expressed alternative isoforms lost more than 60 amino acid residues [8]. Surprisingly, recent transcriptome analysis demonstrated highly alternative mRNA transcripts have similar coding sequences to that of the canonical isoforms, and the sites for protein-protein interaction are usually protected from alternative splicing-mediated removals [12]. Although further functional verification is required to confirm the bioinformatics study, the findings strongly suggested that, the main function of generating multiple protein isoforms by alternative mRNA splicing is not only for generating protein isoforms. Possibilities of putative functions such as mRNA transport, nonsense mediated decay, regulation of gene expression as well as mutational buffering are proposed [13].

Nevertheless, according to Human Gene Mutation Database, it was estimated that about 60% of disease-causing mutations are disturbing proper mRNA splicing [14]. Recent reviews have provided extensive information on splicing mechanisms [15,16,17], and the pathological consequences of mis-splicing that mutations in cis-acting RNA sequence elements and trans-acting splicing factors are described [18,19,20]. However, as splicing dysregulation needs not be directly linked with any genetic mutation, the number of splicing-related diseases shall be substantially underestimated. For example, spontaneous gene mutations in obese subjects with insulin resistance are found but the incidences are rare. Environmental factors play significant roles in their metabolic dysregulation [21,22], potentially by extensive changes in alternative RNA processing [23]. The number and extent of diseases related to splicing dysregulation, which have not been covered by the mutation databases, remain to be explored.

Obesity is a significant leading cause for metabolic diseases, especially for diabetes and cardiovascular problems. The major changes associated with obesity include positive energy balance and activation of immune system [24]. As alternative mRNA splicing is tightly regulated by signaling pathway to cope with the physiological changes [23], diet-induced obesity model can be applied in the investigations of the significance of alternative mRNA splicing in the pathogenesis of obesity. Indeed, significant changes in the expression level of splice variants and splicing factors in association with age and metabolic dysregulation in animal models, as well as in human populations, had already been reported [25,26,27,28,29,30]. Dysregulation of alternative mRNA splicing may stipulate an important driver of ageing process and metabolic diseases.

This review summarizes recent advances on a number of well-known alternative splicing regulated metabolic factors as examples to illustrate the contribution of alternative mRNA mis-splicing in metabolic dysregulation. The potential regulatory mechanism by various splicing factors altering the expression of those splice variants under pathophysiological conditions are included. The possibility by modulation of the expression level and activity of those splicing factors as potential therapeutic targets for obesity-associated metabolic complications will also be discussed.

## 2. Mis-Splicing of Metabolic Factors in Obesity

### 2.1. Insulin Receptor

**Figure 1:**
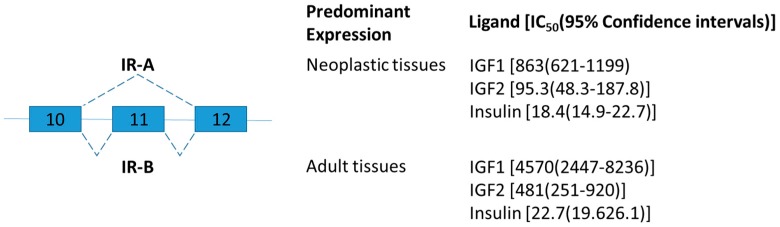
Schematic figure representing insulin receptor (IR) splice variants. Exons 10–12 are represented by boxes. Alternative splicing sites are depicted by connections with dashed lines. The insulin receptor isoform A (IR-A) lacks exon 11 which codes for a 12-amino acid segment present at the C terminal of the alpha chain of the isoform B (IR-B). The ligand binding affinity of human IR-A and IR-B is expressed as IC_50_ values in picomol [35].

Insulin receptor belongs to a subfamily of receptor tyrosine kinases and plays an important role as regulators of cell growth, differentiation, and metabolism [31]. The human insulin receptor exists in two isoforms differing by the presence of exon 11 (Figure 1) [32]. Exon 11, encodes 12 amino acids in the C terminal of insulin receptor and is skipped in a developmental and tissue-specific manner [33]. In brief, insulin receptor type A (IR-A) lacking exon 11 is predominantly expressed during prenatal life for growth and fetal development, and IR-B is predominantly expressed in well-differentiated adult tissues such as the liver for metabolic insulin action [33]. IR-A and IR-B have similar binding affinity for insulin, but different affinity for insulin-like growth factor (IGF)-2 and proinsulin (Figure 1) [34].

Based on the information above, it was hypothesized that insulin sensitivity might be associated with the alteration of insulin receptor isoform expression. Many subsequent studies were performed to explore the correlation between the expression of insulin receptor variants and insulin resistance [36,37,38,39]. As sequence analysis revealed that the region of exons 9 through 12 of rhesus insulin receptor gene is very similar to that of humans, diabetic monkeys were used to explore the potential association between hyperinsulinemia and alternations in the insulin receptor mRNA splicing in 1994 [40]. This study provided the first direct evidence demonstrating that the hyperinsulinemic monkeys have higher levels of IR-A in muscle than those non-hyperinsulinemic controls [40]. The same team further studied the alternative splicing of insulin receptor in liver of normal, prediabetic, and diabetic monkeys. Increase of IR-A level was also observed in the liver of a diabetic monkey, which was significantly correlated with fasting plasma glucose and intravenous glucose disappearance rate [41].

Interestingly, in agreement with the findings in diabetic rhesus monkeys as mentioned above, two recent studies demonstrated that IR-B mRNA variant increased in response to weight loss by either low calorie diet [42] or bariatric surgery in human [43]. They demonstrated that the expression of adipose IR-B is negatively correlated with fasting insulin levels [42,43]. Although conflicting data was reported by other group [44] and the role of insulin receptor isoforms in noninsulin-dependent diabetes mellitus remained elusive, the studies raised the possibility of cellular metabolic status alters the ratio of splice variants. As splicing enhancer and silencer elements are responsible for the alternatively spliced insulin receptor intron 10 and exon 11 [45], several splicing factors (namely hnRNPA1, SF3A, and SFRS7) are proposed to have regulatory role in the exon inclusion of insulin receptor [42]. However, the findings were based on the correlation between the alternative splicing of insulin receptor and the expression of those splicing factors. The detailed molecular mechanism remains to be explored.

Further investigation revealed that Serine and Arginine Rich Splicing Factor 3 (SRSF3; also known as SRP20) play an important role in regulating the insulin receptor exon 11 skipping [45]. Overexpression of SFSR3 results in the inclusion of exon 11, and knockdown of SRSF3 leads to exon 11 skipping in hepatoma cells [45]. In addition, as identified by genomic analysis identified many genes are critical regulators for glucose and lipid homeostasis were mis-spliced in SRSF3HKO liver of SRSF3 liver specific knockout (SRSF3HKO) mice [46]. However, information for the expression level and activity of SRSF3 in obese and diabetes subjects is scarce. Further studies on the regulatory roles of SFSR3 in hepatic glucose and lipid homeostasis under the pathological condition of obese subjects are required.

### 2.2. Leptin Receptor

**Figure 2:**
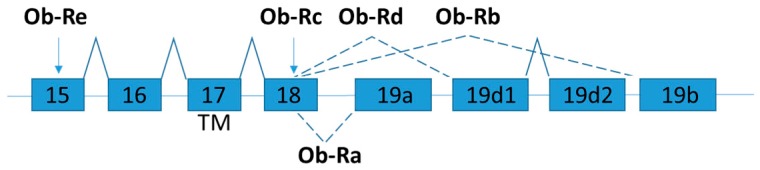
Schematic figure representing leptin receptor (Ob-R) splice variants. Exons are represented by boxes. Alternative splicing sites are depicted by connections with dashed lines. Exon 17 encodes the trans-membrane domain (TM) [59].

Leptin receptor (also known as obesity receptor, Ob-R) is expressed in several isoforms by alternative mRNA splicing (Figure 2) [47]. According to the structural differences, the isoforms are grouped into three classes: namely long, short, and secretory leptin receptors. All Ob-R isoforms have similar N-terminal extracellular ligand-binding domain. The long isoform (named as OB-Rb) is the full-length isoform, which is mainly expressed in the hypothalamus and immune cells and play important roles in energy homeostasis and immunity, respectively [48]. OB-Rb is the only isoform that can fully activate signal transduction. The development of the early obesity phenotype in db/db mice is due to the lacking of Ob-Rb [49].

The short leptin isoforms include Ob-Ra, Ob-Rc, and Ob-Rd. Ob-Ra is the most common isoform which can be found in various tissues (e.g., lung and kidneys) [48]. Although the short leptin isoforms have also the transmembrane domain and a constant box 1 motif at the cytoplasmic domain which binds JAK kinases to activate signal transduction, the main functions of the short isoforms are for internalization and degradation of leptin [50]. Ob-Ra is significantly increased in db/db mice and accounts for their inability to respond to leptin signals [51].

The soluble isoform (Ob-Re or sOB-R) lacks the intracellular and cytoplasmic domains. Ob-Re is suggested to serve as a carrier protein that regulates serum leptin concentration by delaying the clearance of leptin and competitor with membrane receptors for the ligand binding [47]. dbPas/dbPas mice are grossly obese and exhibit hypercholesterolemia and hyperinsulinemia because soluble leptin receptor is absent [52,53]. The leptin and soluble leptin receptor levels in obese and weight-losing individuals are examined by soluble leptin receptor specific ELISA assay [54]. The expression level of soluble leptin receptor is inversely correlated with body mass index (BMI). After weight loss due to gastric restrictive surgery, the expression level of soluble leptin receptor slowly increased to normal level a year after surgery [54]. In contrast to mice, the human soluble OB-R is exclusively generated through proteolytic cleavage of the extracellular domain of membrane-anchored OB-R isoforms [55]. Mutations of leptin receptor gene are rare in humans [56], but polymorphisms in leptin receptor are reported [57]. It solicits further studies in the detailed mechanism on the generation of OB-R isoform and a fully evaluation of its physiological relevance in human [58].

### 2.3. Nuclear Receptor Corepressor

**Figure 3:**
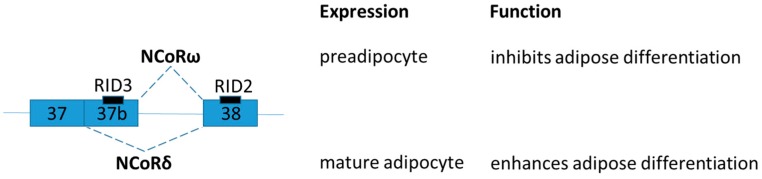
Schematic figure representing nuclear receptor corepressor splice variants NCoRω and NCoRδ. Generation of NCoRω and NCoRδ splice variants are by alternatively splicing at two different 5′ splice donor sites on exon 37. The relative positions of receptor interaction domains (RIDs) coded by exon 37 and 38 are highlighted.

Nuclear Receptor Corepressor (NCoR) is one of the most extensively characterized transcriptional corepressors. NCoR mediates repression of nuclear receptors [thyroid hormone receptor, Liver X receptor (LXR), and peroxisome proliferator activated receptor (PPAR)] by recruitment of chromatin-modifying enzymes (e.g., histone deacetylase 3) [60]. A diverse series of corepressor protein variants of NCoR is generated by alternative mRNA splicing, and different splice variants can exert opposing transcriptional effects [61]. The difference between ω and δ splice variants is by the presence of exon 37, which encodes a third receptor interaction domain (RID) (Figure 3). NCoRω incorporates the exon 37 and has three RIDs [61]. The expression of NCoRω predominates in the preadipocyte, and overexpression of NCoRω inhibits adipose differentiation. In contrast, NCoRδ lacks exon 37 and has only two RIDs [61]. The expression of NCoRδ predominates in the mature adipocyte while its overexpression enhances adipose differentiation [61]. The number of RIDs regulates distinct panels of target genes.

As many NCoR interacting nuclear receptors are playing key roles in the regulation of both glucose and lipid metabolism [61], it was hypothesized that hormonal and nutritive events may regulate the alternative mRNA splicing of NCoR [62,63]. Dexamethasone, a synthetic derivative of a natural hormone that regulates glucose and lipid metabolism, was used to modulate the alternative mRNA splicing of NCoR in both cultured cells and mice [63]. Elevated dietary carbohydrates alter alternative NCoR mRNA splicing. In brief, fructose induced a shift from NCoRω to NCoRδ isoform at the mRNA level in Hepa1-6 hepatocytes, and a shift from NCoRδ to NCoRω was observed in liver tissue of high sucrose fed mice [63]. Although the data is relatively preliminary, the finding strongly supports the idea that hormonal and nutritive events can modulate alternative NCoR mRNA splicing. The precise mechanisms of the upstream nutrient-sensitive signaling pathways that regulate the alternative splicing of NCoR1 shall be further clarified.

To examine the role of switching between NCoRω and NCoRδ in mice, NCoRω splice-specific knockout (NCoRω^−/−^) mice was generated [62]. NCoRω^−/−^ Mice exhibit greatly improved glucose sensitivity that is refractory to diet induced diabetes [62]. It is interesting to note that, as compared with other NCoR1 mouse model listed at Table 1, NCoRω^−/−^ Mice display less severe and distinct phenotypes. Splice-specific knockout mice convincingly indicate that the NCoR variants regulate distinct target genes and hence different phenotypes. These results raise an important concern in determining the roles of genes that encode several variants by alternative splicing in vivo, the particular functions of the alternatively spliced variants could be overlooked by commonly-used whole-gene knockout strategy.

### 2.4. LMNA

**Figure 4:**
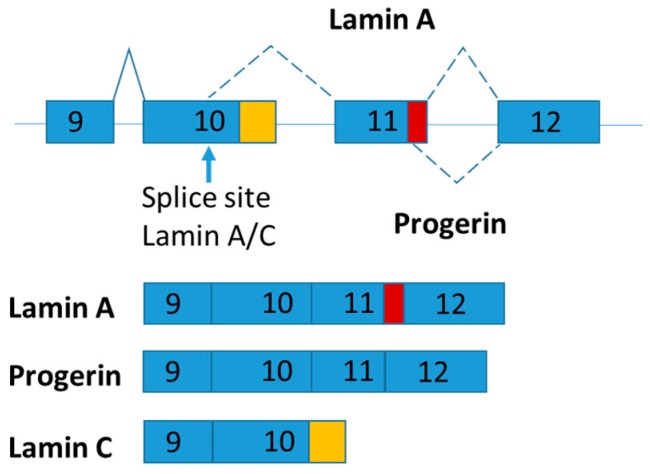
Schematic figure representing LMNA splice variants. The part of exon 11 specific for lamin A is marked in red and the part of 10 specific for lamin C is marked in orange.

LMNA pre-mRNA produces three main isoforms (namely lamin A, progerin and lamin C) by alternative mRNA splicing (Figure 3). Lamins A and C are two major proteins produced from LMNA gene. The LMNA gene has 12 exons and generates lamins A and C by alternative splicing of exon 10 [68]. Both lamins A and C are nuclear intermediate-filament proteins. Progerin is an abnormal truncated version of lamin A protein with deletion of 50 amino acids near the C terminal by mutation (Figure 4). Progerin has also been found in cells and tissues from apparently healthy cells, although its expression is very low [69]. Continuous expression of progerin is suggested contributing to aging associated diseases [69]. Mutations in LMNA gene lead to several diseases called laminphathies (e.g., Emery-Dreifuss Muscular dystrophy and Hutchison-Gilford progeria syndrome) [70]. It was proposed that abnormalities in nuclear structure caused increased susceptibility to cellular damage by the “Mechanical-stress” hypothesis as well as the inappropriate interaction between nuclear envelop and chromatin components as explained the “Gene expression” hypothesis, respectively [71].

Interestingly, lamin C mRNA level was dramatically increased in subcutaneous adipose tissue of obese and type 2 diabetes patients [72]. Given that alternative mRNA splicing of LMNA is highly conserved throughout mammalian evolution, mouse model was used to explore the functions of LMNA isoforms [73]. Knock-in strategy was used to generate LMNA isoform specific expressing mice and found that lamin C and progerin are antagonistic in signaling adipose mitochondrial biogenesis and energy expenditure [74]. In brief, Lmna^LCS/LCS^ mice exclusively expressing the lamin C isoform exhibit obese phenotypes with decreased energy metabolism and mitochondrial activity [74]. In contrast, progerin-expressing mice (Lmna^G609G/+^) present a higher energy metabolism and are lipodystrophic [74].

### 2.5. Lipin-1

**Figure 5:**
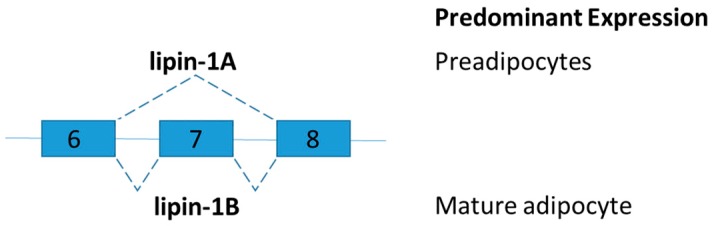
Schematic figure representing lipin splice variants. Lipin-1B differs from lipin-1A by the presence of exon 7. The isoforms of LPIN1 serve distinct functions in adipocytes.

Lipin-1 is an inducible transcriptional coactivator which is required for adipocyte differentiation and lipid metabolism. Two lipin-1 protein isoforms (lipin-1A and lipin-1B) are generated by alternative mRNA splicing of the LPIN1 (Figure 5). Lipin-1B differs from lipin-1A by the presence of exon 7. The isoforms of LPIN1 serve distinct functions in adipocytes [75]. Lipin-1A is mainly expressed in early states of differentiation of preadipocytes, but lipin-1B expression increases with differentiation and is predominant in mature adipocytes. Lipin-1A induces the expression of adipogenic transcription factors peroxisome proliferator-activated receptor gamma (PPARγ) and CCAAT-enhancer-binding protein-alpha (C/EBPα), whereas lipin-1B more effectively induces lipogenic genes such as fatty acid synthase [75]. Studies on lipin-1-deficient mice and tissue-specific lipin-1 transgenic mice showed that lipin-1 is required for adipocyte differentiation. Lipin influences fat mass and energy balance in adipose tissues and skeletal muscle, respectively [76]. To further demonstrate the distinct role of lipin-1A and lipin-1B in vivo and disruption of the relative abundance of lipin-1A and lipin-1B, splice-specific knockout lipin-1A and lipin-1B mice shall be generated.

As mentioned in the introduction, a recent study demonstrated the downregulation of the expression of many RNA processing genes, including SFRS10 (also known as transformer 2 beta homolog TRA2B) in key metabolic organs, liver, and skeletal muscle of obese subjects [28]. Sfrs10 heterozygous mice were generated to explore the role of RNA splicing factor in obesity-related lipogenesis. Interestingly, LPIN1 is a splicing target of SFRS10. Reduced SFRS10 favors the expression of lipogenic lipin-1B [28]. Sirtuin 1 (SIRT1), the key coordinator of the metabolic response to caloric restriction, also plays a regulatory role in the expression of lipin-1B [77]. Ethanol inhibited the expression of SIRT1 which leads to reduced SFRS10 mRNA and protein expression levels in liver [77]. In agreement with previous findings, reduction of SFRS10 increases in expression of lipin-1B in parallel with a decrease in the lipin-1A isoform [28]. Hepatic SIRT1-SFRS10-LIPIN-1A/B axis was explored in the pathogenesis of alcoholic fatty liver disease [77]. Obesity-associated downregulation of SIRT1 in adipose tissues of obese subjects were reported [78,79]. It is interesting to further explore if the SIRT1-SFRS10-LIPIN-1A/B regulatory axis is conserved in adipose tissues.

## 3. The Expression Level of Splicing Factors Altered in Obese Subjects

A number of examples of alternative splicing being changed by hormonal or metabolic signals have been reported [80]. For example, insulin signaling pathway may regulate alternative mRNA splicing via phosphorylation of the splicing factors serine/arginine (SR)-rich proteins and also heterogeneous nuclear ribonucleoproteins (HNRNP). In addition to the post-translational modifications, recent studies demonstrated that expression of several RNA processing genes was altered in various organs of obese subjects [29,81,82]. Further investigation on the dysregulation of splicing machinery components of obesity may provide novel diagnostic and therapeutic tools for this pandemic non-communicable disease.

### 3.1. RNA Binding Protein, Fox-1 Homolog 2

RNA Binding Protein, Fox-1 Homolog 2 (RBFOX2), encodes an RNA binding protein which binds to a conserved element (U)GCAUG stretch in regulated exons or in flanking introns [83], and promotes recruitment of U1 snRNP to the 5′ Splice site leading to inclusion of the alternative exon in the mature transcript [84]. Recent studies demonstrated that RBFOX2 contributes to transcriptome changes under diabetic conditions [81,82]. A genome wide analysis on the alternative splicing profiles of the heart of Type 1 diabetic mouse was changed with a corresponding increase in RBFOX2 protein levels [81]. PKC was identified as regulators of alternative mRNA splicing via the phosphorylation of RBFOX2 [81]. In diabetic heart, the activation of PKC increases the phosphorylation of RBFOX2 which leads to reactivation of fetal alternative splicing programs [81]. Inhibition of PKC activity reduces the steady state levels of RBFOX2 protein [81].

**Figure 6:**
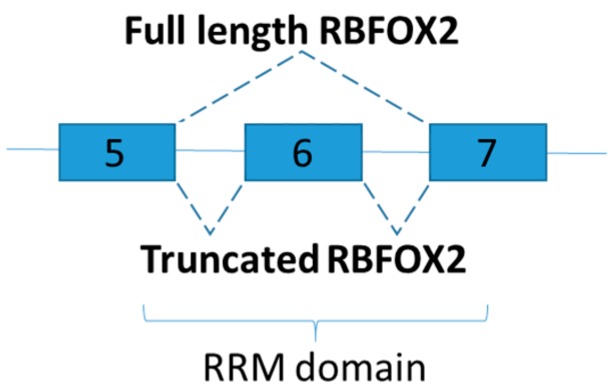
Schematic figure representing RBFOX2 splice variants in the diabetic hearts of mice. As part of RNA recognition motif (RRM) domain is removed, exclusion of exon 6 generates dominant negative version of RBFOX2.

A subsequent investigation demonstrated that RBFOX2 targets more than 70% of mis-spliced pre-mRNAs of the diabetic hearts [82]. Consistent with the study mentioned above [81], RBFOX2 protein levels increased during myocardial differentiation, but alternative splicing activity of RBFOX2 in diabetic hearts is surprisingly low [82]. The phenomenon is explained by the increase of a dominant negative version, instead of the full-length, of RBFOX2 in cardiac tissues of diabetic samples [82]. Dominant negative version of RBFOX2 is generated via exclusion of exon 6, which encodes half to the RNA recognition motif (Figure 6). As a result, dominant negative RBFOX2 has lower RNA binding capability than its full-length RBFOX2, and calcium handling in diabetic hearts is adversely affected by inhibiting RBFOX2 dependent splicing [82]. Although RBFOX proteins facilitate a significant number of the splicing of micro-exons in muscle [3], only five genes relevant to skeletal muscle physiology were found mis-spliced in skeletal muscles of diabetic type 1 despite an elevated RBFOX1 protein levels [78]. A genome wide analysis on the alternative splicing profiles of the skeletal muscle shall be performed. In addition, it is interesting to further explore whether truncated RBFOX2 could be found in human as RRM domain of human RBFOX2 also located on exon 5 to 7 [85].

### 3.2. Neuro-Oncological Ventral Antigen (NOVA) Splicing Factors

Exon skipping is the most common form of HFD-induced mis-splicing [29]. The phenomenon seemed to be adipose tissue specific, as very few HFD-induced mis-splicing were detected in the liver [29]. As NOVA potential binding sites were identified on the mis-spliced pre-mRNAs, NOVA cross-linking immunoprecipitation followed by sequencing (CLIP-seq) was performed to confirm the direct involvement of NOVA proteins in the HFD-induced exon inclusion [29].

NOVA1 and NOVA2 are highly homologous neuron specific RNA-bind proteins and function as alternative splicing regulators. Both NOVA1 and NOVA2 proteins are found in adipocytes [29,86]. HFD treatment decreased NOVA expression in adipose tissues. Adipocyte-specific NOVA deficiency mice were generated to explore the contribution of NOVA to metabolic regulation [29]. Similar to HFD treatment, exon skipping was the most common form of mis-splicing due to NOVA deficiency [29].

Targeting thermogenesis in adipose tissues is a potential strategy treating obesity [87,88,89]. Adipocytes deficient in the NOVA splicing factors displayed increased thermogenesis [29]. The finding is in agreement with the study that demonstrated action of NOVA1 as a brown-adipogenic repressor and manifested the regulation of RNA-binding motif protein 4a (RBM4a) on the expression of NOVA1 [86]. A recent publication showed that body temperature cycles drive rhythmic SR protein phosphorylation to control an alternative splicing program [90]. Change of 1 °C in body temperature is sufficient to induce a concerted splicing switch in a large group of functionally related genes [90]. It is interesting to further explore if alternative mRNA splicing can function as negative feedback system in the regulation of thermogenesis.

## 4. Alternative Splicing as a Therapeutic Target for Obesity

The section above summarizes the emerging evidences of detection of alternative mRNA mis-splicing in obese subjects and discuss the potential of mis-splicing as the root cause of the metabolic dysregulations. Targeting mis-splicing to treat human diseases, gene therapy is one of the approaches to fix the errors in the splicing process raised by mutated cis- and trans-regulatory elements [91]. Therapeutic strategies by small molecule modulators for post-translational modification of splicing factors, antisense oligonucleotides, and trans-splicing have been proposed as potential therapies [92,93]. Several spliceosome inhibitors have been used to treat cancer [94] but exert pronounced cytotoxic effects resulting in abnormal alternative splicing [95,96].

Remarkably, many Food and Drug Administration (FDA) approved marketed drugs, including metformin, affect the alternative splicing machinery [97,98]. Metformin is the first-line anti-diabetic drug which reduces hepatic glucose production, increases intestinal glucose utilization, increases GLP-1 production and alters the composition of the gut microbiota [99]. The proposed underlying molecular mechanisms of metformin are very complicated [100]. A recent study tested the effect of metformin in the treatment of a “spliceopathy-associated” disease, myotonic dystrophy type I (also known as Steinert’s diseases) [98]. Metformin was proposed to act as a modifier of alternative mRNA splicing of a subset of genes mis-splicing in myotonic dystrophy type 1 by activation of AMPK and downregulation of the expression of the RNA-binding protein 3 (RBM3) [98]. It is interesting to explore whether metformin can also fix the pathological mis-splicing in obesity.

In the previous section, we discussed the importance of lamin A/C mRNA level in energy metabolism [72,74]. LMNA luciferase reporter assay was developed for the screening of small molecules modulating SR protein activity [101]. A small molecule named as ABX300 was identified which can abrogate diet-induced obesity by modulating LMNA isoforms via serine and arginine rich splicing factor 1 (SRSF1) in HFD-fed mice [102]. SRSF1 also known as alternative splicing factor 1 (ASF1) and tends to promote exon skipping [101]. A previous study demonstrated that an indole derivative IDC16 inhibits HIV pre-mRNA splicing via targeting SRSF1 [103]. Based on the structure of IDC16, ABX300 was developed. Treatment of ABX300 reversed the mis-splicing induced by HFD potentially by directly binding to and inhibition of SRSF1 [102]. ABX300 also altered the metabolic rate or energy expenditure of mice by promoting the expression of the genes that prevent fat gain or induce fat loss when mice are on HFD [102]. Most importantly, ABX300 did not have any adverse effect on lean mice of normal weight during the study [102]. As the amino acid sequences of human and mouse SRSF1 are 100% identical, it is interesting to explore the translational potential of ABX300 in anti-obesity treatment.

Alternative splicing is regulated by differential splicing factors binding to cis-acting sequences in the pre-mRNA. Antisense oligonucleotides can act as “splice-switchers” by binding to splicing enhancer or splicing silencer elements on the pre-mRNA to modulate alternative splicing [104]. For instance, exon skipping can be induced by using antisense oligonucleotides that bind to the splicing enhancer sequence and create a steric hindrance that blocks the recruitment of stimulatory splicing factor. Vice versa, antisense oligonucleotides binding to splicing silencer elements can promote exon inclusion by preventing the recruitment of negative splicing factors [104].

**Figure 7:**
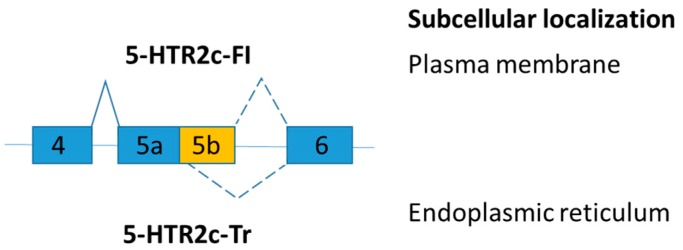
Schematic figure representing serotonin 2C receptor splice variants. The splice variants have very different subcellular distribution.

Serotonin 2C receptor (5-HTR2c) is involved in controlling appetite and food consumption. Alternative exon skipping generates a truncated 5-HTR2c protein isoform [105]. The transcript, including exon 5b, encodes the full-length serotonin 2C receptor (5-HTR2c-FI), but the transcript loss of exon 5b changes the amino acid reading frame and leads to the production of a truncated receptor (5-HTR2c-Tr) (Figure 7). The truncated serotonin 2C receptor (5-HTR2c-Tr) dimerizes with the full-length serotonin 2C receptor (5-HTR2c-FI) and prevents the full-length receptor to reach the plasma membrane [106]. Serotonin enhances satiety and hence reduces food intake [107]. As a result, endoplasmic reticulum retention of 5-HTR2c inhibits serotonin signaling [106]. 

A recent study demonstrated that a snoRNA, SNORD115, regulates the alternative mRNA splicing of serotonin 2C receptor [108] and is missing in patients with Prader-Willi syndrome [109]. The 18 nt oligonucleotide (complementarity against exon 5b and intron 5) promoted the inclusion of exon 5b into the pre-mRNA of 5-HTR2c, and reduced food uptake by increasing the ratio of 5-HTR2c-FI in a mouse model [110]. However, elevated anxiety and hypoactivity with overexpression of 5-HTR2c were reported when intracerebroventricular or carotid injection was used in the delivery of the oligonucleotide [110,111]. Improvement on oligonucleotide delivery is required and potential side effects of the oligonucleotide treatment shall be explored.

**Figure 8:**
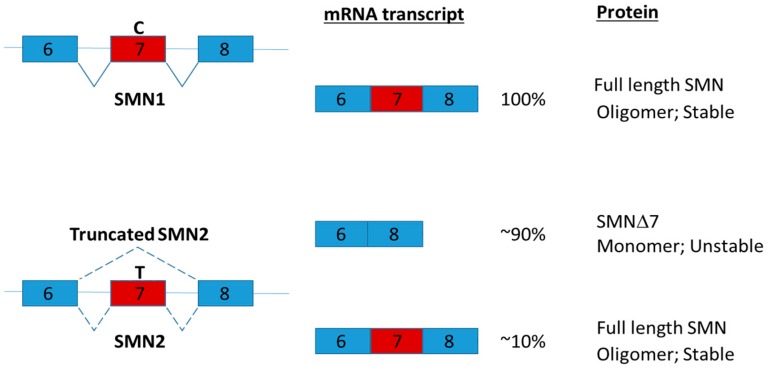
Human SMN1 and SMN2 genes are located respectively in the telomeric and centromeric region of chromosome 5. The single nucleotide difference in exon 7 (C or T as indicated) of SMN1 and SMN2 gene affects their splicing. The single nucleotide change from C to T drastically reduces the efficiency of exon 7 inclusion and increase the production of the truncated mRNAs and proteins [121]. SMN has been shown to self-associate and functions as an oligomer [117]. The stability of SMN protein is highly influenced by oligomerization [119]. The splice variant lacking exon 7 (SMNΔ7) impairs oligomerization leading to rapid degradation of SMN [119]. SMN2 is present in all SMA patients. Splice intervention therapies promote SMN2 exon 7 retention offer a promising approach for SMA therapy by increasing the amount of full-length SMN2 transcript [122].

Intrathecal injection via lumber puncture is one of the promising ways to deliver antisense oligonucleotide to the central nervous system [112]. Nusinersen (marketed as Spinraza; a modified antisense oligonucleotide) was the first approved drug for Spinal Muscular Atrophy (SMA) in December 2016 [113]. SMA is a hereditary disease with global muscle atrophy caused by reduction of survival motor neuron (SMN) protein in spinal cord α-motor neurons [114]. SMN is involved in snRNP assembly, intron retention and DNA damage [115,116]. Reduction of SMN is proposed to disrupt the function of axons by affecting pre-mRNA splicing [117]. In human, two genes SMN1 and SMN2 encode SMN. SMN1 and SMN2 share almost identical amino acid sequences [118]. The critical difference between SMN1 and SMN2 is a single nucleotide difference in exon 7 which play a key role regulating the splicing of the genes [118]. Due to a single nucleotide change, the majority of SMN2 transcripts lack of exon 7 and produce truncated SMN proteins (Figure 8). The truncated SMN proteins are rapidly degraded and decrease the oligomerization efficiency [119]. Nusinersen promotes the inclusion of exon 7 by binding to intron splicing silencer-N1 and hence increasing the production of full length SMN2 protein [120]. Clinical trials in SMA patients have demonstrated that administration of nusinersen to the central nervous system using intrathecal injection significantly improved motor function [113].

As SMN is a ubiquitously expressed protein and metabolic dysregulations associated with SMA have been reported [123,124,125], heterozygous Smn-depleted [126] and severe SMA (Smn^−/−^; SMN2^+/0^) [127] mouse models had been employed to investigate the role of SMN in metabolism. Interestingly, metabolic function of Smn-depleted mice is indistinguishable from the wild type. However, after metabolically challenged with a high-fat diet, Smn(+/−)mice display abnormal localization of glucagon-producing alpha-cells within the pancreatic islets, increased number of insulin-producing beta cells, hyperinsulinemia and increased hepatic glucagon sensitivity [126]. In addition, in the severe SMA mouse model, subcutaneous administration of antisense oligonucleotide, which restored SMN expression, also restored the expression of hepatic IGF1 in SMA mice to normal levels [127]. The findings strongly suggested the importance of SMN in the metabolism of peripheral tissues. It is interesting to further explore the therapeutic potential of nusinersen in the treatment of metabolic diseases such as fatty acid metabolism and glucose homeostasis by systemic administration [128].

## 5. Conclusions

There is a growing interest in the role of alternative mRNA splicing in obese-related metabolic dysregulation. In Table 2, we summarized the splice variants described in this review known to play in metabolic diseases. The examples of alternative mRNA splicing contributed to obese-related diseases cited in this review are far from comprehensive. As recent studies demonstrated, dramatic change of splice variants is detected under different environmental and pathological conditions. Findings provide new information in the development of novel therapeutics by fixing splicing dysregulation. Further investigation on the mechanism of producing and functional consequence of the splice variants are required. The splice-specific KO mice and transgenic mice overexpressing particular splice variant are important tools for investigating unique function of each alternatively spliced variants.

Many approaches have been proposed to manipulate splicing. Use of antisense oligonucleotides is an attractive therapeutic approach for the treatment of diseases related to mis-splicing. The stability and delivery of antisense oligonucleotides are greatly improved by the recent advancement in chemically modifications of the oligonucleotides and delivery methods [104,129]. Numerous antisense oligonucleotides have progressed to human clinical trial for diseases such as muscular dystrophy [130]. While antisense oligonucleotides are still struggling their ways to the clinic, major challenges include off-target effects, efficacy, and immune system activation [131]. Interestingly, many marketed drugs also regulate the alternative splicing machinery, the contribution of those marketed drugs for the treatment of diseases in targeting alternative RNA splicing remains to be explored. Massive researches are still required in the future.

## Figures and Tables

**Table 1:** Summary of the different Nuclear Receptor Corepressor 1 (NCoR1) mouse model.

Mouse Model	Key Metabolic Phenotypes	Proposed Mechanism	Reference
DADm: single amino acid substitution (Y478A) in the Ncor1 DAD domain that is unable to associate with or activate Hdac3	Reduced weight and whole-body fat, Increased oxygen consumption and heat production Increased insulin sensitivity	Increased lipid consumption and obesity-resistant metabolic phenotype	[64]
NCoR ID : contains only 1 RID–N1 and thus would be unable to interact with the thyroid hormone receptor	Reduced body weight with a tendency for lower body fat content Increased oxygen consumption	Increased peripheral sensitivity to thyroid hormone	[65]
Muscle-specific KO	Increased of both muscle mass and of mitochondrial number and activity Reduced LDL cholesterol Improved insulin sensitivity Decreased in the respiratory exchange ratio	Increased muscle quantity and oxidative profile	[66]
Adipocyte-specific KO	Increased adipocyte hyperplasia Reduced inflammation in adipose tissue Increased insulin sensitivity in major metabolic organs (liver, fat and muscle)	Decreased inflammation contributing to the enhancement of insulin sensitivity	[67]
NCoRω^−/−^: NCoRω splice-specific knockout	Increased glucose tolerance Enhanced insulin resistance Increased the size of adipocytes Enhanced liver steatosis Elevated total serum cholesterol level and LDL complexes Reduced in the levels of circulating triglycerides and free fatty acids	Retention of the NCoRω splice variant counteracts prodiabetic physiology in the animals on the HFD	[62]

**Table 2:** Summary of the splice variants described in this review known to play a role in metabolic diseases.

Gene	Changes of Variant Level in Pathological Conditions of Obese Subject	Key Changes in Metabolic Phenotypes	References
Insulin receptor (IR)	Increase IR-A (skipping of exon 11) in the liver	Correlate with fasting plasma glucose and insulin level	[41,42,43]
Leptin receptor (OB-R)	Decrease soluble OB-R *	Correlate with body mass index	[52,53,54]
LMNA	Increase Lamin C in subcutaneous adipose tissue	Correlate with type 2 diabetes	[72]
Lipin-1	Increase Lipin 1-A (skipping of 7) in liver	Cause alcoholic fatty liver disease	[76]
RNA Binding Protein, Fox-1 Homolog 2 (RBFOX2)	Increase truncated RBFOX2 (skipping of exon 6) in heart	Lower calcium handling in diabetic heart	[81]
Serotonin 2C receptor (5-HTR2c)	Increase truncated 5-HTR2c (skipping of exon 5b) in brain	Reduce satiety and enhance food intake	[109]

Remark: * The mechanisms of the soluble OB-R production in human and mouse are different [55].

## Abbreviations

5-HTR2c	Serotonin 2C receptor
HFD	High fat diet
IR	Insulin receptor
KO	Knockout
NCoR	Nuclear Receptor Corepressor
NOVA	Neuro-Oncological Ventral Antigen
nt	Nucleotides
Ob-R	Obesity receptor
RBFOX2	RNA Binding Protein, Fox-1 Homolog 2
SIRT1	Sirtuin 1
SMA	Spinal muscular atrophy
SMN	Survival motor neuron
SR	serine/arginine
SRSF1	Serine and Arginine Rich Splicing Factor 1
SRSF3	Serine and Arginine Rich Splicing Factor 3
LDL	Low-density lipoprotein
